# A case of discrepant laboratory results in samples obtained from a central venous catheter and peripheral veins: when solving a pre-analytical mystery could improve patient care

**DOI:** 10.11613/BM.2022.031001

**Published:** 2022-10-01

**Authors:** Mattia Carini, Moira Micheletti, Giovanni Martellosio, Elisa Caravaggi, Nicola Portesi, Giorgio Biasiotto, Monica Marini, Duilio Brugnoni, Federico Serana

**Affiliations:** 1 Molecular and Translational Medicine Department, University of Brescia, Brescia, Italy; 2 Clinical Chemistry Laboratory, Diagnostic Department, ASST Spedali Civili of Brescia, Brescia, Italy

**Keywords:** blood specimen collection, pre-analytical phase, medical error, central venous catheters, haematology

## Abstract

It is now generally accepted that laboratory errors or inaccurate results are mainly due to deficiencies in the pre-analytical phase. In this report, we describe the case of a 64-year-old male affected by a relapsing follicular lymphoma, who has been treated with chemotherapy through a central venous catheter (CVC). Four different samples were collected alternatively through peripheral venipuncture and CVC sampling. Unexpectedly, the samples collected from the two different sources showed contrasting results, with the presence of unusual macrophage-like cells in the samples obtained from CVC. It was later found that the CVC was displaced into the pleural space. This case report shows how the sampling process can sometimes influence test results and how it can help clinicians identify clinical conditions that have not yet manifested.

## Introduction

Among the various causes of pre-analytical variability, sampling errors are crucial in laboratory medicine. It is well known that they can be attributed to several causes, whether random or systematic (*1*). Since the sampling process consists of a long chain of events, from collection to sample handling and storage, it is not easy to trace the action responsible for altering the process (*2*). Because of this complexity, these errors can sometimes pass unnoticed even when they are well known to clinical pathologists, mainly because of the different multidisciplinary professional figures involved in the chain of action.

The most important consequence is that these errors can often lead to unreliable test results.

For this reason, pre-analytical error must always be considered when ensuring the reliability of a result, especially when a critical laboratory value is involved.

Only when the quality of the pre-analytical phase is improved and standardised through protocols can the accuracy and precision of the data be increased, and the diagnostic or therapeutic process be advanced (*3*, *4*).

In order to standardise the collection procedure, many guidelines and protocols have been established by the scientific community, all of which indicate that blood collection should be performed by peripheral venepuncture (*5*-*7*). Blood collection through a central venous catheter (CVC) may also be considered an acceptable procedure in certain circumstances, for example in critically ill patients when peripheral veins are not readily available, albeit with some caveats. Indeed, Cieslinsky *et al.* showed that the procedure of drawing blood from a CVC can result in reliable data, with no clinically relevant difference compared to peripheral vein sampling, only if the first 10 mL of blood are discarded (*8*). However, this procedure leads to a considerable blood waste that could increase blood transfusion requirements if repeated CVC blood draws were performed over the course of several days (*8*). This loss could be minimized to less than 2 mL if a triple lumen catheter were used, without affecting the quality of the results in comparison to peripheral venous blood sampling (*9*).

Here we report the consequences of alternating blood collection from a central venous catheter (CVC) and from a peripheral vein in a case where the cyclic change of blood collection site resulted in a variability that no one expected.

## Laboratory analyses

At the emergency department of our laboratory at ASST “Spedali Civili” of Brescia two contradictory differential blood counts (DBC) were discussed. They came from two different samplings but they both belonged to the same patient, a 64-year-old male with a medical history of relapsing follicular lymphoma. He was recently admitted to hospital in order to start immunotherapy with the anti CD3/CD19 monoclonal antibody Blinatumumab (Blincyto, Amgen) and for this reason a transjugular venous catheter had been positioned a few days earlier.

Differential blood count was performed using Sysmex XN-20 analysers (Sysmex Corporation, Kobe, Japan) on blood collected in tubes with 1.6 mg/mL K_3_-EDTA in droplet form (Sarstedt, Nümbrecht, Germany), clinical chemistry determinations were performed using Roche Cobas 8000 (Roche Diagnostics, Basel, Switzerland) on blood collected in tubes with 16 I.U./mL Lithium Heparin applied to plastic beads (Sarstedt, Nümbrecht, Germany), coagulation studies were performed using Siemens CS 5100 (Siemens Healthineers, Erlangen, Germany) on blood collected in 0.106 µmol/L Sodium Citrate tubes (Sarstedt, Nümbrecht, Germany).

**Figure 1:**
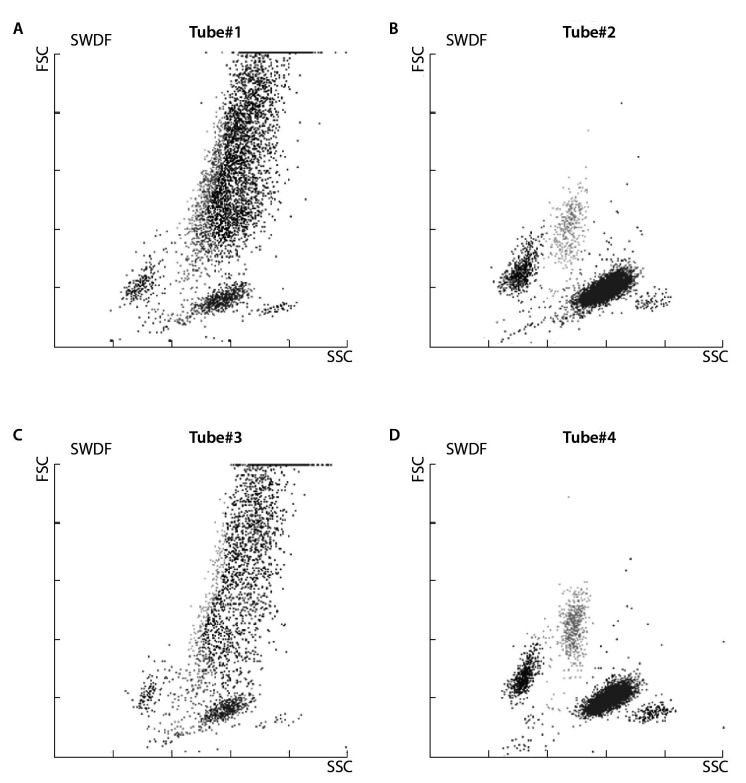
Sysmex XN-20 (Sysmex Corporation, Kobe, Japan) scattergrams of the contrasting tubes. Scattergrams representing the differential blood count cell populations (SWDF) in the samples obtained from CVC (A,C) and obtained from peripheral blood (B,D). A large number of cells (erroneously) labelled by the cell counter as “immature granulocytes” are evident in panels A and C as the darkest dots of the large vertical dot cloud, extending up to the end-scale of the FSC axis. Most of these dots, including the high fluorescence ones, likely represent the macrophages seen in the peripheral smear review. CVC – central venous catheter. FSC – forward scatter.

Three tubes were taken during the first sampling (which we later discovered to be venous blood drawn from a CVC). The main abnormalities were from haematology tube (tube# 1, Table 1): the DBC reported lymphocytopenia (lymphocytes 0.26 x10^9^/L) and severe thrombocytopenia (platelets (PLT) 3 x10^9^/L). Visual inspection of the tube revealed no macroscopic clots and the low platelet count was confirmed by the PLT-F reflex test, which employs a fluorescent dye staining platelet-contained RNA. The scattergrams representing the differential blood count cell populations (SWDF), (Figure 1A) showed a very high percentage of Immature granulocytes (61.5%) with numerous high fluorescence events. As usual in our laboratory, blood film examination was performed. No platelet clumps were observed, but strikingly, some macrophage-like cells were detected.

**Table 1:** Differential blood count results in the four samples

	**Tube #1** **(CVC)**	**Tube #2** **(peripheral vein)**	**Tube #3** **(CVC)**	**Tube #4** **(peripheral vein)**
WBC (x10^9^/L)	5.64	8.39	3.35	6.96
RBC (x10^12^/L)	3.83	3.13	2.27	3.04
Hgb (g/L)	136	110	82	108
Platelets (x10^9^/L)	3	119	33	116
Neutrophils (x10^9^/L)	4.62	7.12	2.98	5.46
Immature Granulocyte (x10^9^/L)	3.47	0.04	2.03	0.03
Lymphocytes (x10^9^/L)	0.26	0.76	0.13	0.65
Monocytes (x10^9^/L)	0.66	0.42	0.20	0.65
Eosinophils (x10^9^/L)	0.07	0.08	0.03	0.18
Basophils (x10^9^/L)	0.03	0.01	0.01	0.02
CVC – central venous catheter. WBC – white blood cells. RBC – red blood cells. Hgb – haemoglobin.				

The remaining two tubes were a clinical chemistry tube and a coagulation one whose blood probably came from the same collection, time and place. The biomarkers tested on the clinical chemistry tube showed only a very high total bilirubin concentration, but direct/indirect bilirubin were not determined because *in vitro* haemolysis interferes with the biochemical method currently in use. No results were obtained from the coagulation tube because the blood was clotted.

During the second sampling, which was carried out on the same day, two more tubes were taken. The DBC from the haematology tube (Tube# 2 in Table 1) showed mild anaemia (haemoglobin (Hgb) 110 g/L), mild thrombocytopenia (PLT 119 x10^9^/L) and a SWDF scattergram (Figure 1B) indicating the absence of immature granulocytes. No macrophage-like cells were detected on examination of the blood film. The results from the coagulation tube were normal.

The conflicting information was passed on to the ward physicians when they were contacted to enquire about some pre-analytical data. The information we obtained was that the first sample was collected from the newly positioned CVC, after discarding the first 10 mL of blood, whereas the second one was taken from a peripheral vein. To explain the severe thrombocytopenia, the clinical haematologists hypothesized an intra-catheter thrombosis. A doppler ultrasound examination was duly performed but no thrombi were detected.

## Further investigation

The same discrepancy occurred the following day when a third and fourth sample were collected, and two more tubes sent to the laboratory.

The results of the haematology tube (tube# 3; Table 1) were similar to those of the first haematology tube, but in addition to the already known lymphocytopenia and thrombocytopenia, unexplained anaemia was found (Hgb 82 g/L). Visual inspection of the tube revealed no macroscopic clots and examination of the peripheral blood smear revealed no platelet clumps. The SWDF scattergram (Figure 1C) again showed a very high percentage of Immature granulocytes (60.6%) with numerous high fluorescence events.

In search of an explanation for this recurrent mystery, a fourth sampling was requested. Once again, the data obtained from the Haematology tube (tube #4) contrasted with those from haematology tube #1 and #3 but were absolutely equivalent to those from haematology tube #2 (Table 1, Figure 1D).

The results were then discussed with the clinical haematologists: on one hand, no obvious catheter-related change was detected (*i.e.,* we did not find any convincing evidence explaining why results were different with CVC use), on the other hand, the only and most apparent difference was the blood collection site. Therefore, in order to minimize the risk of other similar episodes happening, it was decided that from that moment on blood should only be drawn only from a peripheral vein. As a result, contrasting data were no longer obtained in subsequent samples.

## What happened?

Only after an unexpected deterioration in the patient’s clinical condition could the mystery be solved. A few days later, after a severe episode of dyspnoea and low oxygen saturation, a chest X-ray and computed tomography (CT) scan were performed. The CVC was displaced and had migrated into the pleural cavity, resulting in haemothorax and pleural effusion. Only in view of this complication was it possible to explain the pathological results of tube #1 and #3. It is probable that the blood from these samples came directly from the pleural cavity and contained pleural fluid. This fact explains the macrophage-like cells and the contrasting data from the samples taken from the CVC.

## What you can do in your laboratory to prevent such errors

The episode described above is only one of the many events that underline the great influence of the pre-analytical phase on the accuracy and precision of all test results. The improper or inaccurate application of pre-analytical protocols in blood collection can even be dangerous, leading to delays and errors in patient care (*1*, *10*). Although drawing blood from a CVC cannot be considered an error *per se*, this paper shows how the lack of uniformity in the blood collection site can be a source of confusion and can sometimes lead to inaccurate results. Moreover, this incident highlights the important role that the laboratory plays in the diagnostic process. Indeed, this case shows that some contradictory results in the laboratory may hide underlying clinical conditions that may not have manifested yet. This emphasizes the need for closer collaboration between physicians and clinical pathologists.

## References

[1] Lippi G Guidi GC Mattiuzzi C Plebani M. Preanalytical variability: the dark side of the moon in laboratory testing. Clin Chem Lab Med. 2006;44:358–65. 10.1515/CCLM.2006.073 16599826

[2] Plebani M. Errors in Clinical Laboratories or errors in laboratory medicine? Clin Chem Lab Med. 2006;44:750–9. 10.1515/CCLM.2006.123 16729864

[3] Lippi G von Meyer A Cadamuro J Simundic AM. Blood sample quality. Diagnosis (Berl). 2019;6:25–31. 10.1515/dx-2018-0018 29794250

[4] Plebani M. The detection and prevention of errors in laboratory medicine. Ann Clin Biochem. 2010;47:101–10. 10.1258/acb.2009.009222 19952034

[5] WHO World Health Organization (WHO). WHO guidelines on drawing blood: best practices in phlebotomy. Available at: https://www.euro.who.int/__data/assets/pdf_file/0005/268790/WHO-guidelines-on-drawing-blood-best-practices-in-phlebotomy-Eng.pdf. 23741774

[6] Clinical and Laboratory Standards Institute (CLSI). GP41 Collection of Diagnostic Venous Blood Specimens - 7th ed. CLSI document; Wayne PA: 2017.

[7] Simundic AM Bölenius K Cadamuro J Church S Cornes MP van Dongen-Lases EC Joint EFLM-COLABIOCLI recommendation for venous blood sampling. Clin Chem Lab Med. 2018;56:2015–38. 10.1515/cclm-2018-0602 30004902

[8] Cieslinski G Oremek G Klepzig H Jr. Accuracy of blood sampling through central venous lines in intensive-care unit patients. Infusionsther Transfusionsmed. 1993;20:83–8. 10.1159/000222814 8364332

[9] Villalta-García P Lopez-Herranz M Mazo-Pascual S Honrubia-Fernandez T Janez-Escalada L Fernandez-Perez C. Reliability of blood test results in samples obtained using a 2-mL discard volume from proximal lumen of a triple-lumen central venous catheter in the critically ill patient. Nurs Crit Care. 2017;22:298–304. 10.1111/nicc.12220 26487571

[10] Hengeveld RCC Gerards MC Olofsen BE Ridderikhof ML Hakkenberg van Gaasbeek VFHA Leenhouts PA Flushing of an intravenous catheter. Biochem Med (Zagreb). 2019;29:031001. 10.11613/BM.2019.031001 31379463 PMC6610670

